# Effects of Obeticholic Acid Treatment on Primary Human Hepatocytes in a Novel Tri-Culture Model System

**DOI:** 10.3390/cells14130968

**Published:** 2025-06-24

**Authors:** Justin J. Odanga, Sharon M. Anderson, Edward L. LeCluyse, Sharon C. Presnell, Jingsong Chen, Jessica R. Weaver

**Affiliations:** 1 Institute of Regenerative Medicine, LifeNet Health, VA Beach, VA 23453, USA; 2 Research and Development, LifeNet Health, Research Triangle Park, NC 27709, USA

**Keywords:** disease, lipogenesis, inflammation, fibrosis, Obeticholic acid

## Abstract

Metabolic dysfunction-associated fatty liver disease (MAFLD) is a growing health concern worldwide. Human cell-based in vitro culture models that retain disease-relevant phenotypic pathways and responses to assess the efficacy and liability of new therapeutics are needed. Obeticholic Acid (OCA), a Farnesoid X Receptor agonist, has been identified for MAFLD treatment, and clinically shown to have anti-inflammatory and anti-fibrotic effects. In this study, healthy and disease-origin primary human hepatocytes (PHHs) were cultured in TruVivo^®^, an all-human hepatic system for 14 days and treated with OCA to determine its’ effects on lipogenic, inflammatory, and fibrogenic pathways. Decreases in lipogenesis and triglyceride levels were measured in OCA treated healthy and diseased PHHs. Significant decreases in CYP3A4 activity and gene expression were quantified. Macrophage marker expression, pro-inflammatory cytokines and fibrotic markers were lowered in OCA treated diseased PHHs. *CYP7A1* gene expression decreased, while *BSEP* gene expression increased in OCA treated healthy and diseased PHHs. Overall, OCA treatment reduced lipogenic, inflammatory, and fibrogenic markers in diseased PHHs. Differences in the potency and efficacy of OCA against different disease-relevant pathways were observed in healthy and diseased PHHs indicating divergence of key regulatory mechanisms between healthy versus diseased phenotypes.

## 1. Introduction

Metabolic dysfunction-associated fatty liver disease (MAFLD) leading to metabolic dysfunction-associated steatohepatitis (MASH) and cirrhosis is a major health concern with greater than 25% of the general population being affected [1,2]. The severity of liver disease can range from steatosis, MASH, cirrhosis, and ultimately hepatocellular carcinoma [3]. Development of human cell-based in vitro models exhibiting hallmark features and biochemical pathways of disease progression and regression would greatly aid the identification and validation of effective therapeutics for disease treatment. Other key features, including maintenance of a diseased morphology and retention of phenotypic characteristics, such as lipid accumulation and inflammatory and fibrogenic response mechanisms, in a standard convenient culture format over extended culture periods would be of immense value to the biomedical research community. An all-human cell-based in vitro hepatic system known as TruVivo^®^ has been shown to maintain stable albumin and urea production, and Cytochrome P450 (CYP) 3A4 activity for at least two weeks [4,5]. Primary human hepatocytes (PHHs) derived from diseased livers have been used to develop a counterpart diseased system that sustains certain features of a fatty liver disease phenotype, including in vivo-like shifts in hepatocyte functionality, significant basal lipid accumulation, and increased pro-inflammatory cytokine production making it a promising platform for studying differences between PHHs from healthy versus diseased liver tissues [6].

There are numerous different pathways of liver disease that are being considered as drug targets [7,8]. One target being explored is the Farnesoid X Receptor (FXR), a nuclear transcription factor that is activated by bile acids (BAs) [9]. FXR regulates BA synthesis, transport, and reabsorption through its effect on cholesterol 7-α-hydroxylase (CYP7A1), sodium taurocholate co-transporting polypeptide (NTCP), and bile salt export pump (BSEP) [10,11,12]. CYP7A1 is the first and rate-limiting enzyme in the classical BA synthesis pathway [13]. BSEP excretes BAs from PHHs into the biliary system, while NTCP transports BAs from the sinusoidal blood into the hepatocytes [14,15]. FXR plays a critical role in modulating glucose and lipid activity in the liver. Upon BA binding to the FXR, circulating triglycerides and gluconeogenesis from hepatocytes are reduced and downregulated, respectively [16,17].

Obeticholic Acid (OCA) is a small molecule that targets FXR and serves as an agonist with a potency 100 times higher than endogenous BAs [18,19]. OCA has been proposed for use to treat patients with primary biliary cholangitis (PBC), an autoimmune disease that attacks the liver bile ducts [20,21]. OCA was shown to have anti-inflammatory and anti-fibrotic effects by targeting liver sinusoidal endothelial cells (LSECs), kupffer cells (KCs), and stellate cells (SCs) [22]. In addition, OCA has been tested in clinical trials for treatment of liver disease including steatosis, inflammation, and fibrosis [19,23,24,25]. It has been shown to have effects on glucose metabolism and insulin sensitivity [26]. Although OCA has been shown to have these effects, the Food and Drug Administration has yet to fully approve it for treatment of PBC due to the potential of increasing the risk of liver injury in PBC patients with advanced cirrhosis, which resulted in contraindication for prescribing OCA [27]. Furthermore, an increased risk of serious liver injury has been observed in PBC patients without cirrhosis who were not at risk of advanced liver disease. Currently, OCA is being monitored for efficacy and safety [28]. Under these circumstances, it is of critical importance to understand how OCA treatment can affect a specific patient population with varying disease phenotypes in preclinical models, such as TruVivo, ideally before advancing to clinical trials.

The current study uses TruVivo^®^ to determine the effects of OCA on lipogenesis, inflammation, and fibrosis in PHHs from healthy and diseased liver tissues. The aim of this study was to investigate how OCA treatment affects both healthy hepatocytes and hepatocytes in varying disease states. The effect of OCA on hepatocyte functions such as albumin, urea, and CYP450 3A4 activity was measured. In addition, expression of the target genes of FXR, including *NTCP*, *BSEP*, and *CYP7A1*, were examined in healthy and diseased PHHs when treated with OCA. Overall, OCA decreased lipid accumulation in healthy and diseased PHHs. Expression of inflammatory and fibrotic markers was lowered in OCA treated diseased PHHs. However, hepatic function was decreased. An increase in *BSEP* gene expression and a decrease in *CYP7A1* gene expression with OCA treatment was seen in healthy and diseased PHHs. In conclusion, TruVivo^®^ with either healthy or diseased PHHs showed differences in response to OCA allowing for a better understanding of potential heterogeneity in hepatocellular responses that can better mimic the in vivo hepatic microenvironments and phenotypes in liver disease patients.

## 2. Materials and Methods

### 2.1. TruVivo^®^ and OCA Treatment

The approved methods were performed according to the guidelines and regulations of LifeNet Health’s ethics committee. LifeNet Health obtained informed consent for research purposes for all donor tissue. Using the standard NASH (Nonalcoholic steatohepatitis) CRN Scoring system, the liver tissue was histologically assessed by a board-certified liver pathologist to identify the PHHs as healthy or diseased [29]. Tissues with a NAFLD (Nonalcoholic Fatty Liver Disease) Activity Score of ≥4 were identified as “Diseased” and those with a NAFLD Activity Score of ≤1 were identified as “Healthy” (Table 1).

As referenced before, TruVivo^®^ media and cell types were utilized with expected hepatic functional performance [5]. Briefly, PHHs from either healthy or diseased liver tissues were cultured in a 24-well plate under identical conditions for the initial 7 days. Treatment with OCA (0.5 μM) (AdipoGen Life Sciences, San Diego, CA, USA) was initiated on culture day 7 and continued to culture day 14 with daily medium changes [30,31,32]. Due to the heterogeneity in donor lots, there is a possibility of the presence of additional cell types, such as macrophages.

### 2.2. Morphological Assessment and PHH Attachment

Zeiss Observer.Z1 fluorescent microscope (Zeiss, Dublin, CA, USA) and a BX41 microscope (Olympus, Tokyo, Japan) were used to image the cells on the designated days. PHH attachment was determined by the previously described method [5]. Total number of images taken for each condition and time point are stated in the figure legends.

### 2.3. Nile Red Staining

After washing the cells three times with 1X Dulbecco’s PBS (DPBS) (-Ca^++^/-Mg^++^) (Thermo Fisher, Waltham, MA, USA), Nile Red (Abcam, Cambridge, MA, USA) was added (1:500 dilution) on culture day 14. After 15 min at 37 °C, the cells were washed twice with 1X DPBS (-Ca^++^/-Mg^++^). An EVOS FL cell imaging system (Thermo Fisher) on the 20× objective was used to capture images. Total number of images taken for each condition and time point are stated in the figure legends. Quantification of Nile Red fluorescence was done by determining the densitometric fluorescence value (red channel) using ImageJ (version 1.52a). The values were normalized to the determined number of attached PHHs as described above.

### 2.4. Gene Expression

On day 14, RLT buffer (Qiagen, Germantown, MD, USA) was used to lyse the cells. The RNeasy kit (Qiagen) was used to isolate RNA according to the manufacturer’s instructions. A 30 μL volume reaction containing 6 μL PrimeScript RT Master Mix, 20 μL RNA, and 4 μL ddH_2_O was prepared using the PrimeScript RT reagent kit (Takara Bio, Shiga, Japan) to make cDNA. qRT-PCR reactions were prepared using 10 μL QuantiNova 2X SYBR Green Master mix (Qiagen), 2 μL ROX reference dye (1:10 dilution) (Qiagen), 5 μL RNAse/DNase free H_2_O, and 2 μL designated primer set (10 pM). Taqman primers (Thermo) were used for the following genes: *FXR*, *NTCP*, *CYP3A4*, *CD68*, *CD163*, *CYP2E1*, *BSEP*, *CYP7A1*, and *CYP27A1.* Primer sequences (Thermo) used for *Glyceraldehyde-3-phosphate dehydrogenase (GAPDH)*, *Fatty Acid Synthase (FASN)*, *Interleukin-6 (IL-6)*, *Interleukin-10 (IL-10)*, *Cytokeratin-18 (CK18)*, and *Transforming Growth Factor-beta (TGF-β)* are listed in Table 2. PCR amplification was performed on a QuantStudio^TM^ 7 Flex Real-Time PCR System (Thermo Fisher) using the following program: Step (1) 02:00 min at 95 °C; Step (2) 00:05 s at 95 °C; Step (3) 00:10 s at 60 °C. Repeat steps 2 and 3 for 40 cycles. Data was analyzed with QuantStudio^TM^ 7 Flex Real-Time PCR System software (Thermo Fisher) and Microsoft Excel. The housekeeping gene *GAPDH* was used for normalization. Data is shown as C(t) values after normalization to *GAPDH.*

### 2.5. Triglycerides Assay

After washing the cells with 1X DPBS (-Ca^++^/-Mg^++^) (Thermo Fisher), they were lysed in Assay Buffer/Triglyceride Assay Buffer (Abcam). Cell lysates were then incubated for 10 min on ice, centrifuged at 14,000 rpm for 5 min at 4 °C, and supernatants were collected. The fluorometric Picoprobe Triglyceride Quantification Assay kit (Abcam) was performed according to the manufacturer’s instructions to measure triglycerides.

### 2.6. Albumin, Urea, and ELISAs

Cell culture supernatants were collected on the designated days for determining albumin, urea, CK18, and Collagen 1A (COL1A1) levels as described previously [6]. Samples were run in duplicate. ELISA kits were used to determine the level of albumin (Abcam), CK18 (Abcam), and COL1A1 (Abcam). A colorimetric kit (Stanbio, Boerne, TX, USA) was used to determine the level of urea. All kits were performed according to the manufacturer’s instructions.

### 2.7. Assay for Basal CYP3A4 Activity

Baseline CYP3A4 activity (Promega, Madison, WI, USA) was measured using the P450-Glo assay as described previously [6]. A minimum of three wells were used for each condition, and duplicates were run for each sample. After 24 h, the cell culture supernatant was removed, and the cells were washed with DMEM without phenol red (Thermo Fisher). Cyp-Luciferin-IPA stock was added to the cells and incubated at 37 °C for 30 min. After collecting the supernatant, the assay was performed according to the manufacturer’s instructions.

### 2.8. Immunofluorescence

Using Fixation Solution (eBioScience, San Diego, CA, USA), cells were fixed at 4 °C for 30 min. After washing twice with 1X Permeabilization buffer (eBioscience), primary antibody was added and incubated at 4 °C overnight. The following antibodies were used: (1) CD68 at 1:100 (Abcam, ab955); (2) CD163 at 1:100 (Abcam, ab87099); and (3) CK18 at 1:1000 (Abcam, ab24561). After washing the cells two times with 1X Permeabilization buffer, secondary goat anti-mouse IgG Alexa Fluor 488 conjugated antibody (Thermo Fisher) or secondary goat anti-rabbit IgG Alexa Fluor 555 conjugated antibody (Thermo Fisher) was added at a 1:500 dilution at 4 °C for 30 min in the dark. An additional two washes with 1X Permeabilization buffer and one wash with 1X DPBS (-Ca^++^/-Mg^++^) (Thermo Fisher) was done. Fluoromount-G mounting medium with DAPI (Invitrogen, Waltham, MA, USA) was added. A Zeiss Observer.Z1 fluorescent microscope was used to capture the images. Z-stack using a Zeiss ApoTome.2 was used to acquire the images on the 10X objective lens. Total number of images taken for each condition and time point are stated in the figure legends.

### 2.9. 5-(and-6)-Carboxy-2′,7′-Dichlorofluorescein Diacetate (CDFDA) Staining

After washing two times with 1X DPBS (-Ca^++^/-Mg^++^) (Thermo Fisher), DMEM without phenol red (Thermo Fisher) plus CDFDA (5 μm) (Thermo Fisher) was added to the cells on day 14. They were incubated at 37 °C for 20 min. An additional two washes were done with 1X DPBS (-Ca^++^/-Mg^++^), and then DMEM without phenol red (Thermo Fisher) was added to the cells. An EVOS FL imaging system (Thermo Fisher) on the 10X objective was used to capture the images. Total number of images taken for each condition and time point are stated in the figure legends.

### 2.10. Statistical Analysis

Representative donor lots are shown in the images. Values were normalized to the determined number of attached PHHs as described above for albumin, urea, CYP3A4, CK18, and COL1A1 measurements. One-way ANOVA with Tukey or Fisher post hoc testing was used to calculate statistical significance with 95% confidence and * p ≤ 0.05 in MiniTab (MiniTab, State College, PA, USA).

## 3. Results

### 3.1. OCA Treatment and PHH Lipogenesis

**Figure 1:**
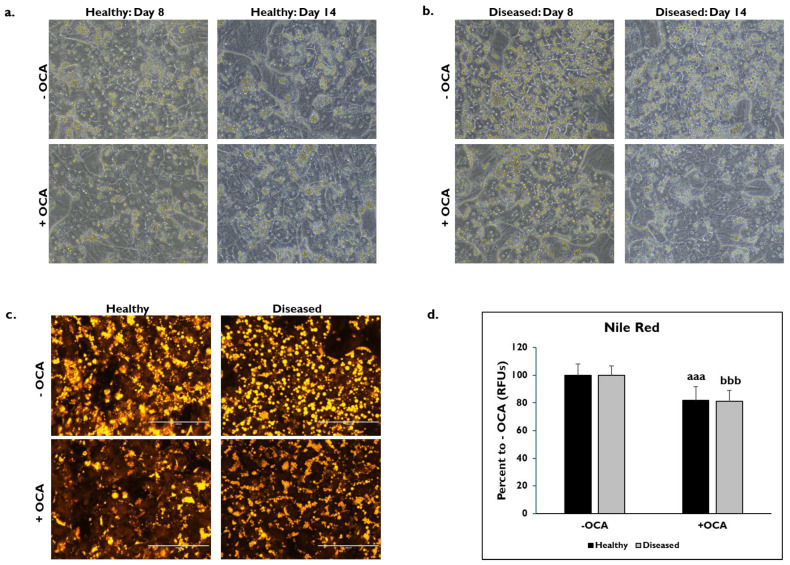
Representative images of (**a**) healthy and (**b**) diseased primary human hepatocytes (PHHs) on days 8 (left column) and 14 (right column) treated without (−) (top row) and with (+) Obeticholic Acid (OCA) (bottom row). PHHs were treated with or without OCA for 24 h (day 8) and 7 days (day 14). Total magnification: 200X. n = 4 healthy donors, n = 4 diseased donors. (**c**) Lipids stained with Nile Red in healthy (left column) and diseased (right column) PHHs when untreated (−) (top row) and treated (+) with OCA (bottom row) on day 14. Total magnification: 200X. n = 2 healthy donors, n = 2 diseased donors. (**d**) Quantitation of fluorescent signal from Nile Red staining on day 14 in healthy (black bars) and diseased (grey bars) PHHs treated without (−) and with (+) OCA. Values have been normalized to untreated PHHs and are shown as percent to untreated PHHs (− OCA). Error bars represent SD. n = 2 healthy donors, n = 2 diseased donors. Nine images were analyzed for each donor. *^aaa^ p ≤* 0.001 to untreated healthy PHHs. *^bbb^ p ≤* 0.001 to untreated diseased PHHs. Significance was determined using ANOVA with Tukey post-hoc testing.

The morphological features between healthy and diseased PHHs treated with OCA and untreated for either 24 h (day 8) or 7 days (day 14) were compared (Figure 1). Healthy and diseased PHHs had hepatocyte morphology with compact, cuboidal shapes and formed colonies with well-defined borders on days 8 and 14 (Figure 1a,b). It appeared that there was increased lipogenesis in the untreated diseased PHHs at both time points compared to the untreated healthy PHHs. Treatment with OCA appeared to decrease lipogenesis in both groups of PHHs. Lipids were stained with Nile Red on day 14 in healthy and diseased PHHs treated with and without OCA with decreased lipid granule size and peripheral localization in diseased PHHs (Figure 1c). There was ~20% decrease in fluorescence in healthy (81.9% ± 10.0 Relative Fluorescent Units (RFUs)) and diseased (81.1% ± 7.8 RFUs) PHHs treated with OCA compared to those not treated (healthy: 100% ± 8.1 RFUs; diseased: 100% ± 6.8 RFUs) (Figure 1d).

**Figure 2:**
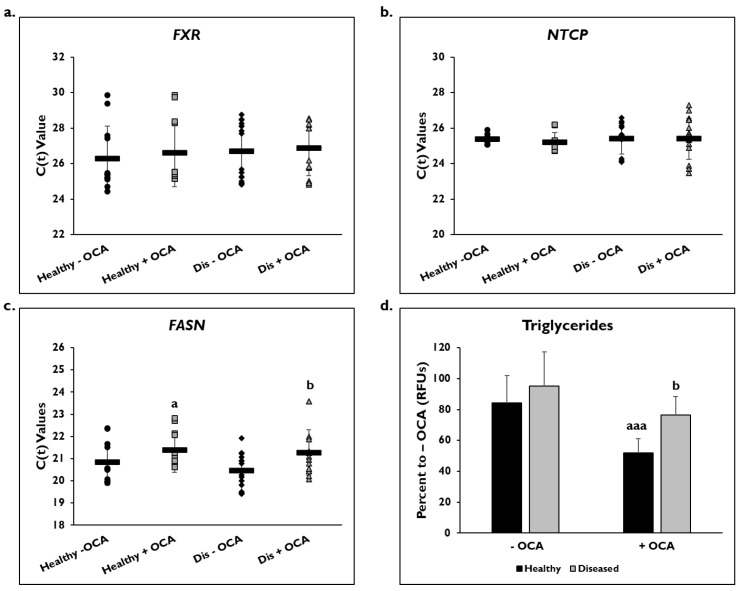
*FASN* gene expression and levels of triglycerides are lowered in healthy and diseased PHHs treated with OCA. Gene expression on day 14 of (**a**) *Farnesoid X Receptor (FXR)*, (**b**) *Sodium Taurocholate Co-transporting Polypeptide (NTCP)*, and (**c**) *Fatty Acid Synthase (FASN)* in untreated healthy primary human hepatocytes (PHHs) (black circles), Obeticholic Acid (OCA) treated healthy PHHs (grey squares), untreated diseased (Dis) PHHs (black diamonds), and OCA treated Dis PHHs (grey triangles). Error bars represent SD. n ≥ 3 healthy donors, n = 4 diseased donors. *^b^ p* ≤ 0.05 to untreated Dis PHHs. (**d**) Levels of triglycerides on day 14 in untreated (−) and OCA treated (+) healthy (black bars) and diseased (grey bars) PHHs. Values have been normalized to untreated PHHs and are shown as percent to untreated PHHs (− OCA). Error bars represent SD. n = 2 healthy donors, n = 2 diseased donors. For each donor, 2 wells were sampled, and each sample was run in duplicate. *^a^ p* ≤ 0.05, *^aaa^ p* ≤ 0.001 to untreated healthy PHHs. *^b^ p* ≤ 0.05 to untreated diseased PHHs. Significance was determined using ANOVA with Tukey post-hoc testing.

Gene expression on day 14 as shown by C(t) values of *FXR* and *NTCP* after normalization to *GAPDH* were measured after treatment with and without OCA for 7 days (Figure 2). There were no significant differences in expression of the *FXR* gene in healthy (26.3 ± 1.8) and diseased PHHs (26.7 ± 1.6) at baseline or after treatment with OCA (healthy: 26.6 ± 1.9; diseased: 26.9 ± 1.5) (Figure 2a). *NTCP* gene expression was also unchanged in both groups before (healthy: 25.4 ± 0.3; diseased: 25.4 ± 0.9) and after OCA treatment (healthy: 25.2 ± 0.6; diseased: 25.4 ± 1.2) (Figure 2b).

*FASN* gene expression as shown by C(t) values after normalization to *GAPDH* was measured to determine whether OCA treatment would influence genes involved in fatty acid synthesis (Figure 2c). In healthy and diseased PHHs, there was a decrease in expression after OCA treatment (healthy: 20.6 ± 0.7 vs. 21.4 ± 1.1; diseased: 20.3 ± 0.7 vs. 21.0 ± 0.9). Both OCA treated healthy and diseased PHHs had a significant change in expression when compared to the untreated PHHs.

When the level of triglycerides was measured, the diseased PHHs (95.1% ± 9.2 RFUs) had a higher baseline value compared to healthy PHHs (84.3% ± 17.7 RFUs) (Figure 2d). The level of triglycerides significantly decreased when OCA was added to the diseased (76.6% ± 11.8 RFUs) and healthy PHHs (51.9% ± 22.1 RFUs); however, the healthy PHHs had a larger decrease (32.4%) compared to the diseased PHHs (18.5%).

### 3.2. OCA Treatment and PHH Functionality

**Figure 3:**
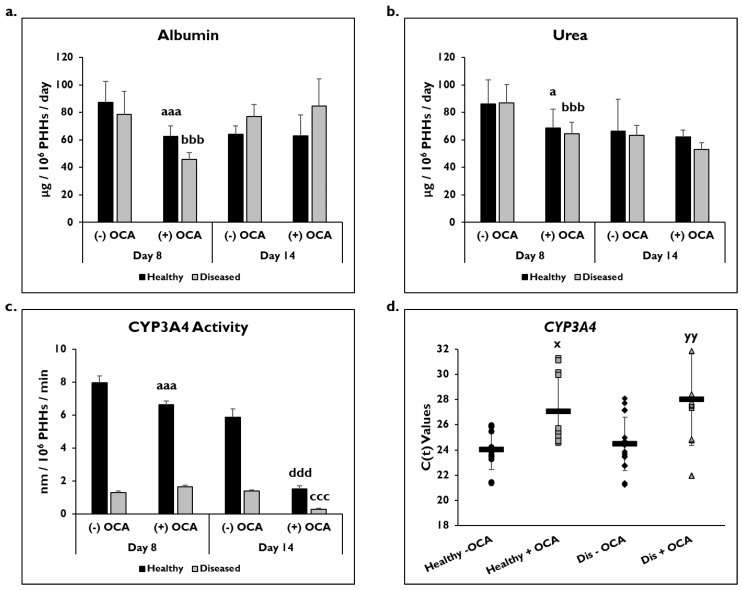
PHH functionality decreases with OCA treatment. Levels of (**a**) albumin, (**b**) urea, and (**c**) Cytochrome P450 (CYP) 3A4 activity in healthy (black bars) and diseased (grey bars) primary human hepatocytes (PHHs) treated without (−) and with (+) Obeticholic Acid (OCA) for 24 h (day 8) and 7 days (day 14). Error bars represent SD. n = 2 healthy donors, n = 2 diseased donors. For each donor, 3 wells were sampled, and each sample was run in duplicate. For CYP3A4 activity, graph depicts representative data from 1 healthy donor and 1 diseased donor. *^a^ p* ≤ 0.05, *^aaa^ p* ≤ 0.001 to untreated healthy PHHs on day 8. *^bbb^ p* ≤ 0.001 to untreated diseased PHHs on day 8. *^ccc^ p ≤* 0.001 to untreated diseased PHHs on day 14. *^ddd^ p ≤* 0.001 to untreated healthy PHHs on day 14. (**d**) *CYP3A4* gene expression on day 14 in untreated healthy PHHs (black circles), OCA treated healthy PHHs (grey squares), untreated diseased (Dis) PHHs (black diamonds), and OCA treated Dis PHHs (grey triangles). Error bars represent SD. n = 4 healthy donors, n = 4 diseased donors. *^x^ p ≤* 0.05 to untreated healthy PHHs. *^yy^ p ≤* 0.01 to untreated diseased PHHs. Significance was determined using ANOVA with Tukey post-hoc testing.

PHH functionality was determined by measuring albumin and urea levels and catabolic activity (CYP3A4) in healthy and diseased PHHs with or without OCA treatment (Figure 3). On day 8, there was a significant decrease in albumin in healthy (62.5 ± 7.6 µg/10^6^ PHHs/day) and diseased (46.0 ± 4.7 µg/10^6^ PHHs/day) PHHs with OCA treatment compared to without treatment (healthy: 87.3 ± 15.2 µg/10^6^ PHHs/day; diseased: 78.6 ± 16.6 µg/10^6^ PHHs/day) (Figure 3a). Although an increase in albumin was seen in the diseased PHHs with OCA treatment (84.5 ± 20.1 µg/10^6^ PHHs/day) compared to no treatment (77.2 ± 8.6 µg/10^6^ PHHs/day) on day 14, it was not significant; however, no increase was seen in the healthy PHHs when OCA was added compared to untreated PHHs (62.8 ± 15.4 vs. 64.0 ± 6.3 µg/10^6^ PHHs/day).

A significant decrease was seen in urea levels on day 8 in healthy (86.3 ± 17.2 µg/10^6^ PHHs/day) and diseased (87.0 ± 13.3 µg/10^6^ PHHs/day) PHHs treated with OCA (healthy: 68.7 ± 13.8 µg/10^6^ PHHs/day; diseased: 64.6 ± 8.4 µg/10^6^ PHHs/day) (Figure 3b). OCA treatment (62.2 ± 5.0 µg/10^6^ PHHs/day) did not affect urea levels in healthy untreated PHHs (66.3 ± 23.3 µg/10^6^ PHHs/day) on day 14. However, the urea levels in diseased PHHs decreased when treated with OCA (53.0 ± 5.2 µg/10^6^ PHHs/day) compared to no treatment (63.5 ± 7.3 µg/10^6^ PHHs/day); however, it was not a significant decrease.

The effect of OCA on CYP3A4 functional activity was measured after 24 h or 7 days of treatment (days 8 and 14, respectively) (Figure 3c). There was a significant decrease in activity in healthy PHHs treated with OCA (6.6 ± 0.2 nm/10^6^ PHHs/min) compared to untreated (8.0 ± 0.4 nm/10^6^ PHHs/min) on day 8. Diseased PHHs exhibited a slight increase in CYP3A4 activity after 24 h of OCA treatment (1.7 ± 0.1 nm/10^6^ PHHs/min) versus no treatment (1.3 ± 0.1 nm/10^6^ PHHs/min). However, by day 14, OCA treatment had a negative impact on CYP3A4 activity with significant decreases seen in both healthy (1.5 ± 0.2 nm/10^6^ PHHs/min) and diseased (0.3 ± 0.06 nm/10^6^ PHHs/min) PHHs when compared to the no treatment group (healthy: 5.9 ± 0.5 nm/10^6^ PHHs/min; diseased: 1.4 ± 0.05 nm/10^6^ PHHs/min). In addition to CYP3A4 activity, *CYP3A4* gene expression after normalization to *GAPDH* was significantly decreased in treated PHHs on day 14 (Figure 3d). OCA treated healthy (27.1 ± 2.7) and diseased (28.0 ± 3.6) PHHs had higher C(t) values after normalization to *GAPDH* compared to untreated healthy (24.0 ± 1.6) and diseased (24.5 ± 2.1) PHHs.

### 3.3. OCA Treatment and Inflammation

**Figure 4:**
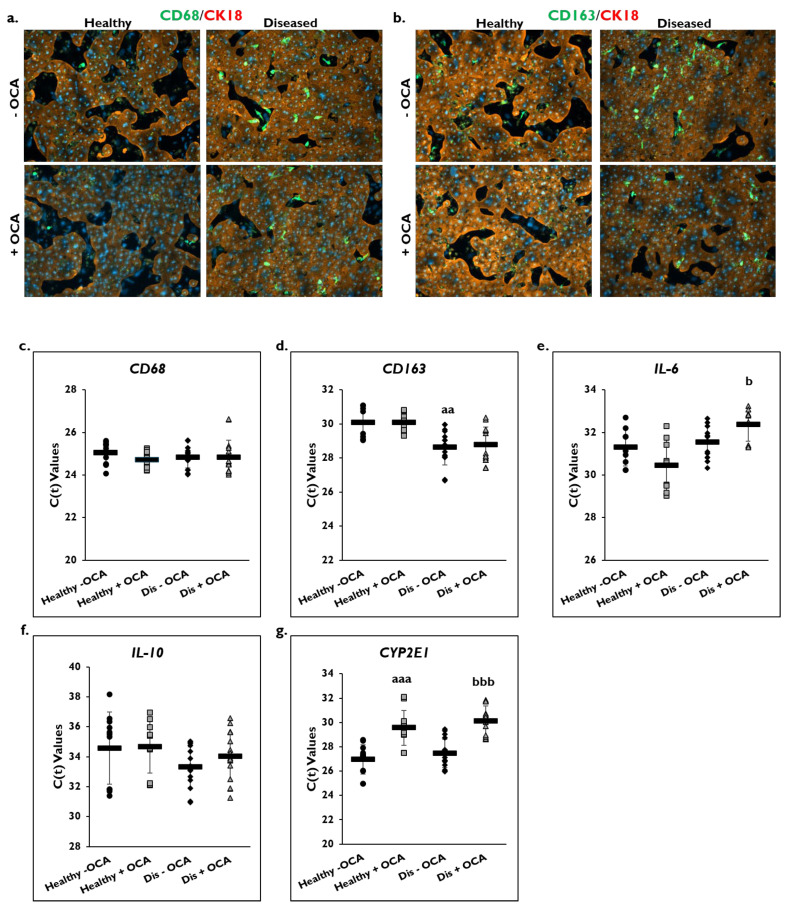
OCA effects localization of macrophage markers and gene expression of pro- and anti-inflammatory markers. Representative images on day 14 of healthy (left column) and diseased (right column) primary human hepatocytes (PHHs) stained for either (**a**) CD68 (green) or (**b**) CD163 (green), Cytokeratin-18 (CK18) (red), and DAPI (blue) when treated without (−) (top row) or with (+) (bottom row) Obeticholic Acid (OCA). Total magnification: 100×. n = 2 healthy donors, n = 2 diseased donors. Gene expression on day 14 of (**c**) *CD68*, (**d**) *CD163*, (**e**) *Interleukin-6 (IL-6)*, (**f**) *Interleukin-10 (IL-10)*, and (**g**) *Cytochrome P450 (CYP) 2E1* in untreated healthy PHHs (black circles), OCA treated healthy PHHs (grey squares), untreated diseased (Dis) PHHs (black diamonds), and OCA treated Dis PHHs (grey triangles). Error bars represent SD. n ≥ 3 healthy donors, n = 4 diseased donors. *^aa^ p* ≤ 0.01, *^aaa^ p* ≤ 0.001 to untreated healthy PHHs. *^b^ p* ≤ 0.05, *^bbb^ p* ≤ 0.001 to untreated diseased PHHs. Significance was determined using ANOVA with Tukey or Fisher post-hoc testing.

The effect of OCA treatment on markers of inflammation was determined in healthy and diseased PHHs by examining the macrophage markers CD68 and CD163 (Figure 4). Cultures of healthy and diseased PHHs stained positive for CD68 and CD163 marker expression on day 14 (Figure 4a,b; Supplementary Figure S1a,b). There appeared to be higher CD68 and CD163 staining in the diseased PHHs compared to the healthy PHHs. OCA treatment did not appear to alter CD68 or CD163 staining in healthy PHHs. However, there appeared to be less staining for both markers when diseased PHHs were treated with OCA.

Gene expression levels for *CD68* and *CD163* on day 14 were measured in OCA treated and non-treated groups in healthy and diseased PHHs (Figure 4c,d). No change in C(t) values after normalization to *GAPDH* for *CD68* gene expression was measured in healthy and diseased PHHs with OCA treatment (healthy: 24.7 ± 0.4; diseased: 24.8 ± 0.8) compared to no treatment (healthy: 25.0 ± 0.5; diseased: 24.8 ± 0.5). This was also true for *CD163* gene expression with no change in C(t) values after normalization to *GAPDH* being measured for treated (healthy: 30.0 ± 0.6; diseased: 28.8 ± 1.0) versus untreated healthy (30.1 ± 1.0) and diseased (28.6 ± 1.0) PHHs. It should be noted that diseased PHHs had a significantly higher C(t) value than healthy PHHs meaning diseased PHHs had greater *CD163* expression.

OCA treatment on expression of the *IL-6* and *IL-10* genes as shown by C(t) values after normalization to *GAPDH* on day 14 was determined (Figure 4e,f). An increase in *IL-6* gene expression was measured in healthy PHHs treated with OCA (30.4 ± 1.2) compared to no treatment (31.3 ± 0.9). However, OCA treatment (32.4 ± 0.8) significantly decreased *IL-6* gene expression compared to untreated (31.6 ± 0.8) diseased PHHs. This decrease was seen for *IL-10* gene expression in diseased PHHs treated with OCA (34.0 ± 1.6) compared to diseased PHHs that did not receive treatment (33.3 ± 1.4); however, it was not significant. No change in C(t) values for *IL-10* gene expression were measured in healthy PHHs whether they were treated (34.7 ± 1.8) or not (34.6 ± 2.4).

*CYP2E1* gene expression as shown by C(t) values after normalization to *GAPDH* was measured in OCA treated healthy and diseased PHHs (Figure 4g). OCA treatment significantly decreased expression of *CYP2E1* in healthy (29.6 ± 1.4) and diseased (30.1 ± 1.3) PHHs versus untreated healthy (27.0 ± 1.2) and diseased (27.5 ± 1.2) PHHs.

### 3.4. OCA Treatment and Fibrogenic Markers

**Figure 5:**
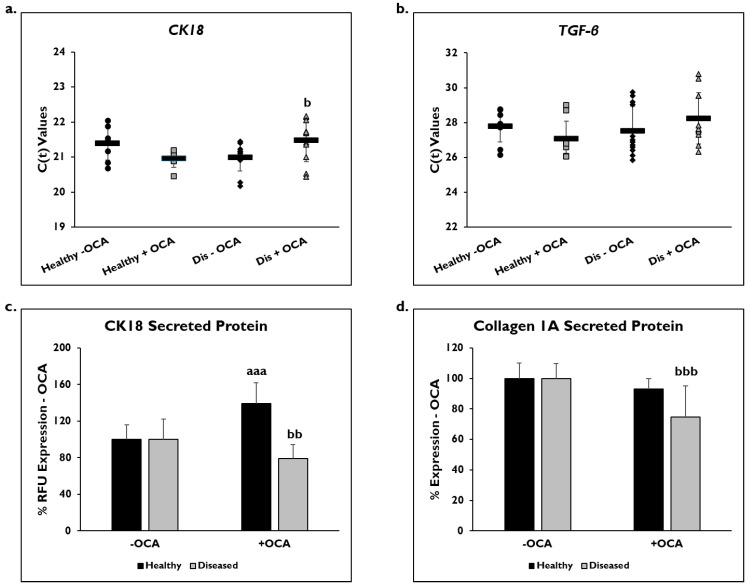
OCA treated diseased PHHs have decreased fibrotic marker expression. (**a**) *Cytokeratin-18 (CK18)* and (**b**) *Transforming Growth Factor-beta (TGF-β)* gene expression on day 14 in untreated healthy primary human hepatocytes (PHHs) (black circles), Obeticholic Acid (OCA) treated healthy PHHs (grey squares), untreated diseased (Dis) PHHs (black diamonds), and OCA treated Dis PHHs (grey triangles). Error bars represent SD. n = 2 healthy donors, n ≥ 2 diseased donors. For each donor, 2 wells were sampled, and each sample was run in duplicate. (**c**) CK18 and (**d**) Collagen 1A secreted protein on day 14 in healthy (black bars) and diseased (grey bars) PHHs treated without (−) and with (+) OCA. Values have been normalized to untreated PHHs and are shown as percent decrease to untreated (− OCA). Error bars represent SD. n = 2 healthy donors, n = 2 diseased donors. For each donor, 3 wells were sampled, and each sample was run in duplicate. *^aaa^ p* ≤ 0.001 to untreated healthy PHHs. *^b^ p ≤* 0.05, *^bb^ p* ≤ 0.01, *^bbb^ p* ≤ 0.001 to untreated diseased PHHs. Significance was determined using ANOVA with Tukey post-hoc testing.

Markers of fibrogenic response, CK18, TFG-β, and COL1A1, were examined to determine if OCA influenced their expression (Figure 5). Gene expression was measured on day 14 in healthy and diseased PHHs that were treated with or without OCA. C(t) values after normalization to *GAPDH* for *CK18* gene expression were significantly decreased in diseased PHHs treated with OCA (21.6 ± 0.7) compared to untreated diseased PHHs (21.0 ± 0.4) (Figure 5a). OCA treatment did not seem to influence *CK18* expression (21.1 ± 0.6) in healthy PHHs compared to untreated (21.4 ± 0.5). When *TGF-β* gene expression was measured, untreated diseased PHHs had higher expression levels (27.5 ± 1.4) compared to OCA treated (28.2 ± 1.5) diseased PHHs (Figure 5b); however, this increase was not significant. There was an increase in *TGF-β* expression in healthy PHHs with OCA treatment (27.1 ± 1.0) versus no treatment (27.8 ± 0.9), a trend similar to *CK18* gene expression.

Secreted CK18 protein and COL1A1 were measured in OCA treated healthy and diseased PHHs on day 14 (Figure 5c,d), and quantified values were normalized to untreated PHHs. There was a significant decrease in CK18 protein in diseased PHHs treated with OCA (79.7% ± 15.5 RFUs) compared to untreated diseased PHHs (100% ± 22.5 RFUs). However, in healthy untreated PHHs (100% ± 16.1 RFUs), there was a significant increase in CK18 secreted protein when treated with OCA (139.1% ± 22.6 RFUs). COL1A1 significantly decreased in diseased PHHs (74.7% ± 20.6 RFUs) treated with OCA compared to untreated diseased PHHs (100% ± 9.7). A similar decrease was observed in healthy PHHs with OCA treatment (93.0% ± 6.9 RFUs) versus untreated PHHs (100% ± 10.3 RFUs); however, it was not significant.

### 3.5. Effects of OCA on BA Synthesis Pathways

**Figure 6:**
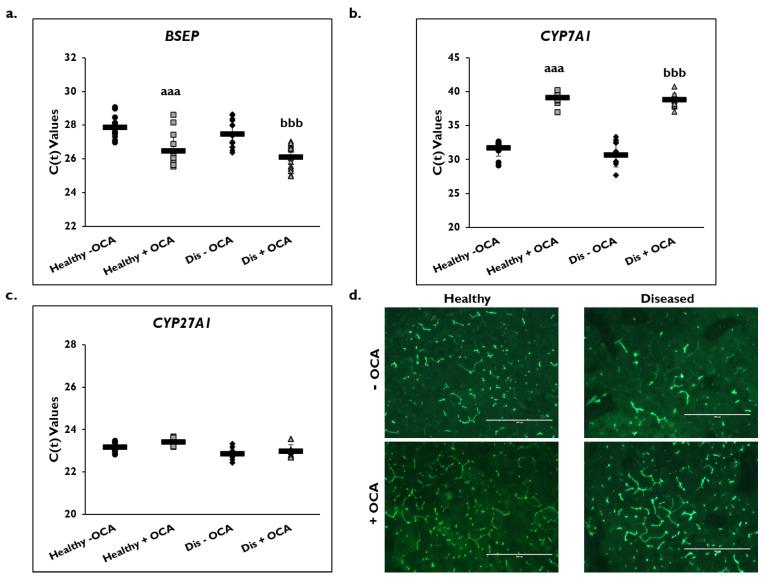
Expression of bile acid synthesis genes are altered in healthy and diseased PHHs treated with OCA. Gene expression on day 14 of (**a**) *Bile Salt Export Pump (BSEP)*, (**b**) *Cholesterol 7-α-hydroxylase 7A1 (CYP7A1)*, and (**c**) *Cytochrome P450 (CYP) 27A1* in untreated healthy primary human hepatocytes (PHHs) (black circles), Obeticholic Acid (OCA) treated healthy PHHs (grey squares), untreated diseased (Dis) PHHs (black diamonds), and OCA treated Dis PHHs (grey triangles). Error bars represent SD. n ≥ 3 healthy donors, n ≥ 3 diseased donors. *^aaa^ p* ≤ 0.001 to untreated healthy PHHs. *^bbb^ p* ≤ 0.001 to untreated diseased PHHs. (**d**) Representative images on day 14 of bile canaliculi staining in healthy (left column) and diseased (right column) PHHs treated without (−) (top row) and with (+) (bottom row) OCA. Total magnification: 100×. n ≥ 3 healthy donors, n ≥ 3 diseased donors. Significance was determined using ANOVA with Tukey post-hoc testing.

Expression of several genes in the BA synthesis and transport pathways were examined on day 14 (Figure 6). *BSEP* gene expression significantly increased in healthy (26.5 ± 1.1) and diseased (26.1 ± 0.7) PHHs with the addition of OCA compared to untreated PHHs (healthy: 27.9 ± 0.7; diseased: 27.5 ± 0.8) based on C(t) values after normalization to *GAPDH* (Figure 6a). Gene expression of *CYP7A1* significantly decreased in OCA treated healthy (39.1 ± 1.0) and diseased (38.8 ± 0.9) PHHs compared to untreated PHHs (healthy: 31.7 ± 1.2; diseased: 30.7 ± 1.7) (Figure 6b). Similar *CYP27A1* gene expression was measured in the treated (23.4 ± 0.2) versus untreated (23.2 ± 0.3) healthy PHHs and in the treated (23.0 ± 0.3) disease compared to the untreated diseased (22.9 ± 0.3) PHHs (Figure 6c).

Because OCA is an FXR agonist that is used to treat PBC, its effect on the BA synthetic and transport pathways was examined (Figure 6d). The formation of bile canaliculi was examined by immunofluorescence microscopy using CDFDA on day 14 in healthy and diseased PHHs when treated with and without OCA. There appeared to be an increase in the anatomizing of the bile canaliculi network when healthy and diseased PHHs were treated with OCA. This effect was observed to be more prominent in the diseased versus healthy PHHs treated with OCA.

## 4. Discussion

When OCA was evaluated in TruVivo^®^, there was a significant decrease in lipogenesis and triglyceride levels in healthy and diseased PHHs. Although there was no change in *FXR* gene expression, FXR activation is thought to have occurred through its pathway effects including decreased triglyceride levels potentially through Sterol Regulatory Element Binding Protein 1c, which is involved in fatty acid synthesis in the liver [33]. In addition, there was a significant decrease in *FASN* gene expression, which is regulated by Sterol Regulatory Element Binding Protein 1c, in the healthy and diseased PHHs [34,35]. Overall, OCA treatment significantly reduced the accumulation of lipid vesicles in healthy and diseased PHHs in TruVivo^®^.

OCA had varying effects on albumin and urea levels and CYP3A4 activity. It increased albumin in diseased treated PHHs, though not significantly, and decreased urea levels throughout the culture period in diseased PHHs. A review of the Phase II and Phase III clinical trials for OCA found no significant changes in albumin levels in the OCA treated groups compared to placebo treated groups, similar to these current findings [36]. Interestingly, FXR appears to play a role in urea synthesis by acting as a transcriptional regulator of enzymes involved in the urea cycle [37,38]. Studies using FXR-knockout mice treated with OCA showed increased expression of proteins that regulate generation of urea and glutamine synthesis suggesting that therapeutics such as OCA that activate FXR may promote ammonium clearance in MASH patients [38]. The decrease in urea levels in diseased PHHs with OCA treatment could be from OCA activating FXR and promoting ammonia clearance in order to create homeostasis and a normal physiological state in these PHHs, similar to the current condition of the healthy PHHs treated with OCA.

OCA significantly impaired CYP3A4 activity in healthy PHHs but increased activity in diseased PHHs on day 8. However, by day 14, the CYP3A4 activity in healthy and diseased PHHs was significantly decreased. Corresponding gene expression of *CYP3A4* on day 14 showed a significant decrease. This result is similar to what has been previously observed in studies done by Nørgaard and others when examining OCA in advanced in vitro systems [39,40]. However, these data do not correspond to the clinical findings, although CYP3A4 suppression was suggested at the higher dose of OCA [41]. Upregulation of CYP3A4 expression has been observed after FXR activation, which was suppressed by the FXR agonist GW4064 through a mechanism mediated by a small heterodimer partner [42,43]. Further studies examining the mechanism of regulation would need to be performed to obtain a better understanding of CYP3A4 upregulation and/or suppression by OCA through FXR in vitro.

The impact of OCA treatment on inflammatory responses in healthy and diseased PHHs was examined. The two macrophage markers CD68 and CD163 were examined. CD68 is categorized under the M1 phenotype and thought to express pro-inflammatory cytokines, such as IL-6 and Monocyte chemoattractant protein-1 [44]. The M2 phenotype includes CD163 expressing macrophages that secrete anti-inflammatory cytokines such as IL-10 [44]. Staining of these macrophage markers appeared to decrease in diseased PHHs treated with OCA but not in healthy PHHs under the same conditions. However, gene expression of *CD68* and *CD163* did not show a significant change when healthy and diseased PHHs were treated with OCA. Notably, there was a decrease in gene expression of *IL-6* and *IL-10* in cultures of diseased PHHs treated with OCA. These results are consistent with previous work showing the inhibition of murine liver KCs by OCA after activation with lipopolysaccharide or Tumor Necrosis Factor-α [22]. In that study, *Monocyte Chemoattractant Protein-1* and *IL-10* gene expression in KCs were decreased with OCA treatment after stimulation but measured no changes in expression without stimulation, a condition that may be similar to the healthy PHHs shown. Additional studies showed a decrease in gene expression of the IL-6 signaling pathway after treatment with OCA in PHHs [35]. In our current studies, decreases in cytokine expression in diseased PHHs may be attributable to decreases in activation of the NLR family pyrin domain containing 3 inflammasome in macrophages because of OCA treatment [45]. Preliminary unpublished FLOW cytometry data suggests a level of cell heterogeneity including the inclusion of functional macrophages in the PHH donor lots. The diseased PHH donor lots showed a higher level of CD68^+^ expressing cells versus healthy donor lots. The level of CD163^+^ cells was similar between the two types of donors. In our previously published data, changes in CD68^+^ and CD163^+^ marker expression were observed between diseased fibrotic versus healthy PHHs after enriching in either high or low Percoll post-thaw [46]. It should be noted that the PHHs were not under stress or pharmacological stimulation. Future studies will determine the degree of heterogeneity between healthy and diseased donor lots. This could also explain why macrophage gene expression was unchanged, yet macrophage marker expression and cytokine expression were affected with OCA treatment.

The effects of OCA on *CK18* gene expression and protein levels, a marker of fibrogenic responses, were determined. There was a decrease in diseased PHHs treated with OCA compared to untreated diseased PHHs. Interestingly, normal PHHs showed an increase in both *CK18* gene expression and secreted protein. A decrease in COL1A1 secreted protein was also seen in diseased PHHs with OCA treatment versus nontreated diseased PHHs. *TGF-β* gene expression had a similar result with increased expression in healthy OCA treated PHHs and a decrease in expression for diseased OCA treated PHHs. Several studies showed decreases in *TGF-β* gene expression and genes in its pathway in cirrhotic rat livers and PHHs treated with OCA [22,35]. A study examining liver biopsies from the Phase III REGENERATE trial found OCA to have antifibrotic effects [23,47]. A better understanding of how OCA is working to reduce fibrogenic responses and outcomes has been performed using several murine models [22,48,49]. These murine models show OCA inhibiting hepatic SC proliferation and activation, which occurred through an increase in BA accumulation. Inhibition of CYP7A1 was also detected in some of these models providing further evidence of OCA’s influence on BA accumulation [48,49]. Overall, this decrease in SC activity results in lessening of fibrosis. Based on our data, a similar mechanism of how OCA works to improve fibrosis may be occurring in the diseased PHHs, but further studies are needed.

Gene expression of *CYP7A1*, *CYP27A1*, *BSEP*, and *NTCP* were examined due to OCA’s effects on PBC. While *CYP7A1* gene expression was significantly reduced in healthy and diseased PHHS treated with OCA, *BSEP* gene expression was significantly increased in healthy and diseased PHHS that received OCA treatment. There did not seem to be an effect on *CYP27A1* gene expression, the initiator for the alternative BA synthesis pathway, in the healthy and the diseased PHHs treated with OCA. There was no change in *NTCP* gene expression in the absence of exogenous BAs in the medium, which may be expected due to there being no change in *FXR* gene expression or its’ influence on *NTCP* expression [50]. The effect of OCA treatment on lowering triglyceride levels in healthy and diseased PHHs could be the result of decreased *CYP7A1* and increased *BSEP* expression.

In addition to changes in gene expression, healthy and diseased PHHs treated with OCA appeared to have increased bile canaliculi networks. This effect seemed to be greater in the diseased PHHs that had less bile canaliculi formation compared to healthy PHHs; however, with the addition of OCA, these networks became more extensive in the diseased PHHs. It has been found that increased FXR expression plus the addition of a FXR agonist increased BA secretion due to increased BSEP expression [51]. It may be that these results in TruVivo^®^ indicate that OCA activation of FXR is having a choleretic effect by increasing both BSEP expression and translocation of subcellular storage vesicles, leading to increased BA transport and more extensive bile canalicular network formation in the diseased PHHs. Because healthy PHHs inherently possess higher capacity to transport BA excretion, there is less of a requirement for an increased bile canaliculi network capacity and function [52].

Overall, TruVivo^®^ demonstrates greater efficacy compared to other co-culture models due to testing diseased hepatocytes in this model system. Several co-culture models, including sandwich culture, have shown anti-fibrotic and anti-inflammatory effects by culturing healthy hepatocytes in a lipogenic medium that induces a diseased state [35,53]. However, this model system has the capability of culturing diseased hepatocytes for 14 days without a collagen overlay, without the addition of NPCs, and uses standard cell culture medium to determine the effects of the OCA.

In conclusion, healthy and diseased PHHs maintained in TruVivo^®^ and treated with OCA showed decreased lipogenic capacity that is consistent with known clinical effects. Gene expression of *FASN* and triglyceride levels were lowered. Little to no changes were seen in albumin levels with OCA treatment; however, urea levels and *CYP3A4* gene expression and activity significantly declined. OCA treated diseased PHHs showed lessening of inflammatory and fibrogenic response pathways. Decreases in *IL-6*, *IL-10*, *CK18*, and *TGF-β* gene expression were measured plus lower CK18 and COL1A1 protein levels in diseased PHHs with OCA treatment. Healthy and diseased PHHs treated with OCA had decreased *CYP7A1* gene expression and increased *BSEP* gene expression. Although OCA treatment influenced inflammation and fibrosis, this effect was dependent on disease designation. Furthermore, these disease-like features were increased in healthy PHHs treated with OCA, suggesting uncharacteristic mechanisms are happening with treatment. This study highlights the potential danger of therapeutic treatment when not fully understood and the inconsistencies in response to treatment across a diseased population. The use of these preclinical model systems, such as TruVivo®, where both healthy and diseased PHHs can be studied will allow for increased understanding of molecular mechanisms regarding disease states and ultimately connect preclinical models to clinical outcomes. These preclinical models also have the potential to more effectively address regulatory concerns from agencies such as the FDA before entering into clinical trials. TruVivo^®^ has the potential to further distinguish important differences in disease origin signaling pathways and other targets that can represent both healthy and diseased phenotypes.

## 5. Conclusions

In summary, the advantages of this model system, coupled with the flexibility and convenience of employing standard plate formats, suggest that this novel platform represents a promising new tool for testing the effects of new drug therapeutics for liver disease.

## Figures and Tables

**Table 1:** Characteristics of Donors.

Donor (Lot #)	Age	Sex	BMI	NAS Score	Steatosis Score	Lobular Inflammation Score	Hepatocyte Ballooning Score	Fibrosis Stage	Health Category
16117	41	F	30	0	0	0	0	0	Healthy
1814680	52	F	23	0	0	0	0	0	Healthy
1919613	60	M	26	0	0	0	0	1–2	Healthy
2117885	23	F	23.7	1	1	0	0	0	Healthy
2021736	50	F	25.6	6	3	1	2	0	Diseased
2111401	28	M	21.8	5	3	0	2	1	Diseased
2116167	51	M	29.8	4	1	1	2	1	Diseased
2222437	30	M	26.8	4	1	1	2	1	Diseased

**Table 2:** Sequences of Primers.

Primer Name	Forward Sequence (5′-3′)	Reverse Sequence (5′-3′)
*GAPDH*	GGTCACCAGGGCTGCTTTTA	GGATCTCGCTCCTGGAAGATG
*FASN*	AAGGACCTGTCTAGGTTTGATGC	TGGCTTCATAGGTGACTTCCA
*IL-6*	ACAACCTGAACCTTCCAAAGA	TCAGCAGGCTGGCATTT
*IL-10*	GGCTACGGCGCTGTCATCGATT	GCATTCTTCACCTGCTCCACGG
*CK18*	GGCATCCAGAACGAGAAGGA	AGTGCTCCCGGATTTTGCT
*TGF-* *β*	TTCCCTCGAGGCCCTCCTA	GCCGCAGCTTGGACAGGATC

## Data Availability

The datasets presented in this article are not readily available because the data is part of an ongoing study. Requests to access the datasets should be directed to jessica_weaver@lifenethealth.org.

## Supplementary Materials

The following supporting information can be downloaded at: https://www.mdpi.com/article/10.3390/cells14130968/s1, Figure S1: Additional representative images of inflammatory marker expression after OCA treatment.

## Abbreviations

The following abbreviations are used in this manuscript:

BA	Bile acids
BSEP	Bile salt export pump
CDFDA	5-(and-6)-Carboxy-2′,7′-Dichlorofluorescein Diacetate
COL1A1	Collagen 1A
CYP	Cytochrome P450
CYP7A1	Cholesterol 7-α-hydroxylase
CK18	Cytokeratin-18
DPBS	Dulbecco’s PBS
FASN	Fatty Acid Synthase
FXR	Farnesoid X Receptor
GAPDH	Glyceraldehyde-3-phosphate dehydrogenase
IL-6	Interleukin-6
IL-10	Interleukin-10
KC	Kupffer Cell
LSEC	Liver Sinusoidal Endothelial Cell
MAFLD	Metabolic Dysfunction-Associated Fatty Liver Disease
MASH	Metabolic Dysfunction-Associated Steatohepatitis
NAFLD	Nonalcoholic Fatty Liver Disease
NASH	Nonalcoholic steatohepatitis
NTCP	sodium taurocholate co-transporting polypeptide
OCA	Obeticholic Acid
PBC	Primary biliary cholangitis
PHH	Primary Human Hepatocyte
RFU	Relative Fluorescent Unit
SC	Stellate Cell
TGF-β	Transforming Growth Factor-beta
