# Supplementary material for: Effects of Obeticholic Acid Treatment on Primary Human Hepatocytes in a Novel Tri-Culture Model System
Source: Cells. 2025 Jun 24;14(13):968. doi: 10.3390/cells14130968 (PMC12249178; doi:10.3390/cells14130968)
Supplement: Supplementary file 1 [file cells-14-00968-s001.zip › cells-3640675-supplementary.pdf]

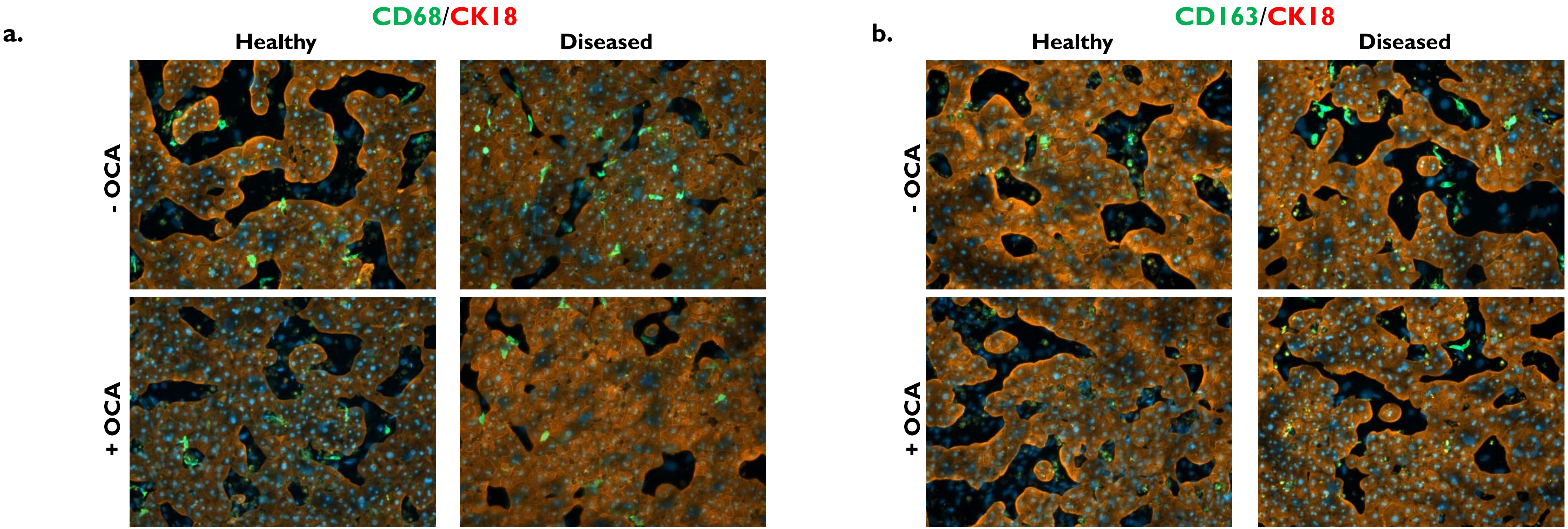


**Supplementary Figure 1.** Additional representative images on day 14 of (a) CD68 (green) and (b) CD163 (green) plus Cytokeratin-18 (CK18) (red) and DAPI (blue) staining in healthy (left column) and diseased (right column) primary human hepatocytes (PHHs) treated with (+) Obeticholic Acid (OCA) versus no treatment (-). Objective magnification: 10X. n = 2 healthy donors, n = 2 diseased donors.
